# Cocktail vaccine for the management of *Hyalomma anatolicum* and *Rhipicephalus microplus*

**DOI:** 10.3389/fimmu.2024.1471317

**Published:** 2024-11-19

**Authors:** Haranahally Vasanthachar Manjunathachar, Binod Kumar, Balasamudram Chandrasekhar Parthasarathi, Gajanan M. Chigure, Buddhi Chandrasekaran Saravanan, Muthu Sankar, Darasaguppe Ramachandra Harish, José de la Fuente, Srikanta Ghosh

**Affiliations:** ^1^ Entomology Laboratory, Division of Parasitology, Indian Council of Agricultural Research (ICAR)- Indian Veterinary Research Institute (IVRI)-Izatnagar, Bareilly, Uttar Pradesh, India; ^2^ Division of Animal Biotechnology, Indian Council of Agricultural Research (ICAR)-Indian Veterinary Research Institute (IVRI)-Izatnagar, Bareilly, Uttar Pradesh, India; ^3^ SaBio, Instituto de Investigación en Recursos Cinegéticos (IREC), Consejo Superior de Investigaciones Científicas (CSIC), Universidad de Castilla-La Mancha (UCLM)-Junta de Comunidades de Castilla-La Mancha (JCCM), Ciudad Real, Spain; ^4^ Department of Veterinary Pathobiology, Center for Veterinary Health Sciences, Oklahoma State University, Stillwater, OK, United States; ^5^ Indian Veterinary Research Institute (IVRI)-Eastern Regional Centre, Kolkata, West Bengal, India

**Keywords:** RNAi, ferritin2 (FER2), tropomyosin (TPM), cocktail vaccine, *Hyalomma anatolicum*, *Rhipicephalus microplus*, cross-bred cattle

## Abstract

**Introduction:**

Globally, ticks rank second only to mosquitoes as vectors of deadly pathogens affecting humans and first in transmitting animal pathogens, presenting a significant challenge to human wellness and sustainability of livestock-based industries. Traditional tick control via chemical acaricides impacts on the environment and has led to the emergence of multi-acaricide-resistant tick populations. Use of immunoprophylactic, along with other components of integrated tick management, holds the potential to mitigate tick infestations in a sustainable manner. To control multi-species tick infestations, the concept of a cocktail vaccine comprising of more than one antigens has emerged as a viable solution due to the inconsistent efficacy of single antigen-based immunization protocol.

**Methods:**

In this study, a dual antigen cocktail immunization protocol was developed targeting ferritin2 (FER2) and tropomyosin (TPM) proteins, which are associated with ticks’ essential cellular and physiological functions, like blood iron homeostasis and muscle contractions.

**Results:**

Dual gene silencing of FER2 and TPM genes in *Hyalomma anatolicum* resulted in a 75.3% reduction in infested ticks, a 95.4% decrease in egg masses, and a complete loss of egg hatching when compared to control ticks. Microscopically, an altered ovarian cellular architecture, marked by vacuolation and reduced nucleus-to-cytoplasmic ratio were noted in the gene knocked down ticks. An immunization with cocktails of 300 µg dose of each protein, rHaFER2 and rHaTPM was standardized in a rat model and was used to immunize cross-bred (*Bos indicus* x *B. taurus*) male cattle with Montanide ISA 50V2 adjuvant on days 0, 28, and 49. A significant (*p* < 0.001) IgG and IgG2 antibody response was observed in the immunized animals with high IgG levels sustained until day 119 post-primary immunization, showing a 4.1-fold increase over the pre-immunization period. The animals were challenged with larvae and adults of *H. anatolicum* and larvae of *Rhipicephalus microplus*. Immunization with the cocktail antigen resulted an efficacy of 70% and 76% against *H. anatolicum* larvae and adults, respectively, and 54% against *R. microplus* infestations. Compared to single-antigen immunization, the immunization with cocktail antigens demonstrated higher protection against *R. microplus* and *H. anatolicum* ticks. The results advance the development of cocktail vaccines to control multiple tick species.

## 1 Introduction

Ticks are obligate blood-feeding ectoparasites of vertebrate hosts and act as vectors for various viral, bacterial, protozoans, rickettsial, and fungal pathogens impacting human and animal health globally (1). Ticks are second only to mosquitoes as vectors of human diseases and are the primary transmitters of many arthropod-borne diseases to livestock and companion animals (2). In the tropical and subtropical regions of the world, *Rhipicephalus microplus* and *Hyalomma anatolicum* ticks are considered as the most economically important species, significantly impacting the growth of the cattle industry by direct damage to animals and by transmitting a number of fatal pathogens viz., *Babesia* spp., *Anaplasma* spp., *Theileria* spp (3, 4). Besides, these tick species are also transmitting fatal zoonotic pathogens causing Indian tick typhus (ITT), Kyasanur Forest Disease (KFD), and Crimean-Congo Hemorrhagic fever (CCHF), which are spreading to different parts of the globe (5–8). Globally, annual economic losses from ticks and tick-borne diseases (TTBDs) are estimated to be between $22-30 billion USD, highlighting their upward negative impact on human and animal health worldwide (9). Nevertheless, various attributing factors, *viz*, globalization, intensified transboundary trade activities, movement of hosts, climate change, and anthropogenically induced changes in land usage patterns, have significantly contributed to the rise in TTBDs worldwide (4, 10).

Tick management is mainly based on repeated acaricide applications with limited efficacy and cause numerous effects on hosts, the environment, and public health (11, 12). Misuse of different acaricide classes have led to the emergence of resistant tick populations in various parts of the world (12–15). Besides resistance, indiscriminate use of chemical acaricides has increased environmental pollutants and contaminated animal byproducts with chemical residues (16). Among alternative tick control strategies, the immunological approach is considered one of the most promising, being target-specific, eco- friendly, and economically sustainable (17, 18). However, to date, only two vaccines (TickGARD™ and GAVAC™) have been commercialized for reducing *R. microplus* infestations in cattle, offering limited cross-protection due to the tick genetic diversity (17, 19–21). Since then, numerous new tick protective antigens have been identified and tested against homologous challenges, with efficacy ranging from 47-90%. However, these antigens have shown limited cross-species protective efficacy (18, 22, 23). Dowall et al. (24) suggested that zoonotic tick-borne diseases like CCHF could be managed by developing and using commercially viable, potent cross-protective anti-tick vaccines as emphasized previously by other tick researchers (25–27). Earlier, Willadsen (28) opined that combining two or more antigens in a cocktail form would significantly increase the overall efficacy of immunization compared to immunization by a single antigen. Different multi-antigens immunization strategies such as immunization with cocktail of antigens, co-immunization, and immunization using chimeric antigens were advocated. Among the different strategies, the immunization using a cocktail of antigens of targeted tick species provided better efficacy than other strategies (29–32). However, the identification of suitable antigens for the development of multicomponent vaccines conferring protective efficacy against multiple tick species remains a major challenge. Nonetheless, researchers are striving hard to develop protocol for inducing substantial humoral and/or cell-mediated immune response against multiple tick infestations following immunization. Few studies have shown that host immunoglobulins can cross the tick gut to the hemolymph and cells, leading to antibody-mediated cell lysis (33, 34). These findings have increased the possibility of targeting both intracellular and secretary molecules in vaccine development.

Blood intake is necessary for each tick to complete molting and female ticks to complete egg laying and fertility. Consequently, genes involved in the physiological processes of tick blood-feeding were targeted for anti-tick vaccine development (35). Accordingly, two molecules namely, Ferritin2 (FER2), gut specific secretory protein involved in iron homeostasis and expressed in all the tick stages and Tropomyosin (TPM), an actin associated salivary protein, play vital roles in muscle contraction by modulating the binding of myosin and highly conserved between tick species and other invertebrates were chosen in the current study (36–39). RNAi studies and immunization trials conducted by different tick research groups using these two antigens alone have shown promising anti-tick effects mainly by disrupting blood feeding, oviposition, and fecundity of ticks fed on immunized animals (36, 37, 39, 40). Taking the leads from the previous experiments, the current study is focused on developing a cocktail vaccine formulation for better efficacy against homologous and heterologous challenge infestations.

## 2 Materials and methods

### 2.1 Ticks and experimental animals

The acaricide susceptible *Hyalomma anatolicum* (IVRI-II) and *Rhipicephalus microplus* (IVRI-I) strains maintained in the Entomology laboratory, Division of Parasitology, Indian Veterinary Research Institute (IVRI) were used as reference material. Cross bred bovine calves (*Bos indicus* X *B. taurus*) above six months of age were maintained at the large animal experimental shed of the division. The calves were maintained as per the approved guidelines laid down by the Committee for the Purpose of Control and Supervision of Experiments on Animals (CPCSEA) [No.F.26-1/2015-16/J.D(R)]. Similarly, healthy 1 to 1.5 kg New Zealand white rabbits and adult male Wistar rats (200–250 g) were maintained in the small animal house of the division. Rabbits were used for rearing of *H. anatolicum* (41), while rats were used for the standardization of immunization dose. Rats and rabbits were maintained as per the guidelines of Institute Animal Ethics committee (IAEC) [No. F. 26-1/2015/JD (R)].

### 2.2 Double gene silencing and its impact on biology of ticks

Double gene silencing was carried out in *H. anatolicum* ticks as per the method of Nijhof et al. (42). Gene-specific primers were designed with consensus T7 promoter at the 5’ end for *in vitro* transcription and synthesis of *Ha-FER2* (KT924235) and *Ha-TPM* (KU297197) genes of *H. anatolicum*. The Luc dsRNA was used as RNAi control. Cloned bacterial plasmid containing *FER2* and *TPM* genes of *H. anatolicum* and luciferase from vector pGEM-luc (Promega, USA) were used as a template for amplification. The amplified products were purified before *in vitro* transcription and purification of dsRNA using MEGAscript^®^ RNAi kit (Ambion, USA). Furthermore, the number of molecules per microliter was quantified using web based OligoCalc software (version 3.27) based on the molecular weight of dsRNA. Samples were aliquoted, labeled and stored at -20°C for downstream processing. A group of 7–10 days old 100 unfed females (4 replications of 25 each) of IVRI–II strain ticks were inoculated with 0.5–1 μl of dsRNA each (5-10 X 10^11^ molecules/µl) of *FER2* and *TPM* genes using a 10 µl Hamilton syringe with 34G RN needle as described previously (39, 43). Similarly, an equal number of ticks were inoculated with Luc dsRNA and designated as control groups. After inoculation, each tick was gently removed from the adhesive tape and placed in a 100 mL wide bottom container and the ticks were activated by blowing air in the container. The ticks were then incubated at 85% RH and 28°C temperature for 24 hours and examined for their vigor and only active females were released along with equal number of male ticks on cross bred bovine calves using ear bags (41). Ear bags were examined 48 hours post-release of ticks. The effects of double gene silencing of *H. anatolicum* were calculated from three test replications based on the percentage of ticks successfully engorged, rejected, unable to engorge (UTE), mean engorgement weight and mean egg masses between the treated and control group as per the method of Kumar et al. (43). Post RNAi, genes were quantified using fully engorged and UTE ticks collected after 72 h and 96 h of release with the control group as well as normal ticks collected at same intervals. Fold change in gene expression between control and inoculated ticks was compared by one-way ANOVA and the Tukey tests against control at *p*> 0.05 of significance.

### 2.3 Processing of post RNAi *H. anatolicum* ticks for histological studies

Post RNAi, one batch (n = 25) of engorged ticks were dissected to collect the ovaries and processed as per the laboratory standardized protocol (44). Briefly, ticks were fixed on the dissection plate filled with paraffin after anesthetizing using thermal shock at 4°C for 10 min. The dorsal cuticle was gently lifted from the anterior end and the attachments from visceral organs were removed under a dissecting microscope (Olympus microscope BX53, Japan). Then ovary was removed and transferred to chilled Phosphate Buffered Saline (PBS), washed twice and then transferred to the fixative solution of 4% paraformaldehyde (Sigma-Aldrich, USA) for 24 hours. Tissues were cut into small pieces of 4-6 mm size and then dehydrated in ascending strength of ethanol (70, 80, 90 and 95%) for 15 minutes each at room temperature. Infiltration and embedding of ovarian tissue were done with JB-4 Embedding Kit (Sigma-Aldrich, USA) and transferred to polyethylene embedding BEEM^®^ (Polysciences, Inc., USA) capsules filled with resin containing a catalyzer. After polymerization, tissues were sectioned (3 μm) using ultra-microtome (Leica EM UC7, Vienna) and stained with hematoxylin and eosin (H & E).

### 2.4 Expression of rHaFER2 and rHaTPM recombinant proteins

The HaFER2 open reading frame (ORF) devoid of signal peptide and complete HaTPM proteins were expressed in *E. cloni* ^®^ 10 G chemically competent cells (Lucigen, USA) using the pRham-N-His-SUMO-Kan expression vector (Lucigen, USA). Polyhistidine tagged (6× Histag) containing rHaFER2 and rHaTPM proteins were purified under denaturation conditions by metal chelate affinity chromatography (Ni-NTA chromatography). The purified denatured proteins were refolded by dialysis against gradually decreasing concentrations of urea and concentrated using cut-off device (Pall filter, UK) and stored at -20°C in the presence of cocktail protease inhibitor at the rate of 10 μl/ml (Amresco, USA). The reactivity of the rHaFER2 and rHaTPM proteins was determined through western blotting as described previously (39, 45).

### 2.5 Determination of immunization dose

The dose standardization protocol used for rHaTPM antigen was adopted (45). Briefly, rats (Wistar, adult male, 200–250 g in weight) were divided randomly into six groups of five rats in each. The rHaFER2 and rHaTPM were mixed (1:1 ratio) with Montanide™ ISA 50 V2 ready to use adjuvant (SEPPIC, France) by homogenization. The animals of groups I, II, and III were immunized with 50, 100 and 150 μg of each protein in a cocktail on 0^th^, 14^th^ and 28^th^ day. At the same time, animals of groups IV, V, and VI received equal volume of adjuvant mixed with PBS and were designated as the control group. Antibody titers were monitored by indirect ELISA and compared between the immunized and control group of animals by ANOVA (*p*< 0.05). For ELISA, antigen concentration and other variables were optimized through the checkerboard method. The primary antibodies were diluted serially from 1:200 to 1: 25,600 to determine antibody titer in each group of animals. The ELISA plates (F96 Maxisorp, Nunc, Roskide, Denmark) were coated with 100 µl of carbonate buffer (pH 9.6) containing a specific concentration of rHaFER2 and rHaTPM and stored at 4°C overnight. After washing with PBS-0.05% Tween 20 (PBS-T), each well was blocked with 100 μl of 5% skimmed milk in PBS-T at 37°C for 2 h. The plates were incubated with 100 μl/well of rat sera diluted serially starting at 1:200 with blocking buffer and incubated at 37°C for 2 h. The ELISA plates were washed thrice with PBS-T and 100 μl of 1:10000 dilution of HRP-conjugated rabbit anti-rat IgG (Sigma-Aldrich, USA) was loaded in each well and incubated at 37°C for 2 h. After washing thrice, a 100 μl of o-phenylenediamine dihydrochloride (OPD) (Pierce, USA) in citrate buffer, pH 5.0 was loaded in each well and incubated for 15 min at RT. The optical density (OD) was read at 492 nm in an ELISA reader (Tecan Sunrise, Austria).

### 2.6 Identification of native FER2 and TPM proteins in the developmental stages by western blotting

The presence of FER2 and TPM proteins in different developmental stages of *H. anatolicum* was determined using specific anti-FER2 and anti-TPM sera raised previously in rats immunized with the respective recombinant proteins and were available in the laboratory. Briefly, egg, larvae, unfed nymph, engorged nymph, unfed adult, partially fed females and fed males of IVRI-II strain were homogenized in cold 0.15 M PBS, 1 mM disodium EDTA, pH 7.2, containing cocktail protease inhibitors, filtered, sonicated and centrifuged at 15,000 rpm for 60 min at 4°C as described earlier (46). The supernatants were designated as antigens of the respective stages. The protein concentration of the antigen was estimated by Bradford assay. The individual extract was resolved in 10% SDS-PAGE and transferred to a Nitrocellulose membrane (NCP). The membranes were processed sequentially by incubating with a primary antibody using rat anti-ferritin and anti-tropomyosin sera (1:500 dilutions) followed by peroxide-conjugated rabbit anti-rat IgG (1:2000 dilution, Sigma) and the chromogenic substance, DAB (diaminobenzidine). The reaction was stopped by washing the membranes thoroughly with distilled water. The image was captured and stored.

### 2.7 Immunization and challenge study

Eight cross-bred bovine calves aged six to seven months were randomly divided in two groups of four animals in each group. A cocktail of antigens was then prepared by emulsifying both the antigens thoroughly with an equal volume of Montanide ISA 50V2 adjuvant (SEPPIC, France) to prepare a milky-white water-in-oil emulsion with a final concentration of 50 µg of each antigen/ml. The cocktail of antigens was administered via deep intramuscular injection into the gluteal muscles on days 0, 28, and 49 at 2 mL/animal/dose. The control group of animals were immunized with 2 ml of PBS emulsified with the same adjuvant as the placebo. Two weeks after the second booster, all the animals were challenged with 15 pairs (1:1 male and female) of 7-10 days old unfed adults of *H. anatolicum* to each ear pinna using the ear bag method as described by Ghosh and Azhahianambi (41). Four weeks after the first challenge, each animal was challenged again with 10-12 days old larvae hatched from 50 mg eggs of both *H. anatolicum* and *R. microplus.* The ear bags were regularly checked and dropped larvae and adults were kept in BOD incubator, maintained at 28 °C and 85% relative humidity (RH). Engorged larvae were maintained till molting to nymphs and engorged females till the end of egg laying. Immunization efficacy was measured based on the reduction of number of challenged larvae, molting and rejection percentage, engorgement weight and adult fertility rate (20). The immunization efficacy was calculated as per the following formula:

Against larvae


E (%)=100 [1−(CRT X CRM)]

$$\begin{array}{c} \text{Percentage reduction in challenged larvae }[\text{DT }\left(\%\right)\\ =100\;\left(1-\text{NTV}/\text{NTC}\right)]; \end{array}$$

$$\begin{array}{c} \text{Percentage reduction in molting of engorged larvae }[\text{MO }\left(\%\right)\\ =100\;\left(1-\text{MLI}/\text{MLC}\right)]; \end{array}$$

$$\begin{array}{c} \text{Percentage efficacy of antigen against larvae }\{\text{E }\left(\%\right)\\ =100\;\left[1-\left(\text{CRT}\times \text{CRM}\right)\right]\}; \end{array}$$

where, NTV & NTC: number of larvae dropped from the immunized and control group of animals, respectively. MLI and MLC: number of engorged larvae molted to nymphs from immunized and control group of animals, respectively.


CRT=NTV/NTC and CRM=MLI/MLC

CRT: CRT is the reduction in the number of larvae (NTV/NTC).

CRM: CRM is the reduction in number of engorged larvae molted to nymphs (MLI/MLC).

Against adults


DT%=100 (1−NTV/NTC),

DT: reduction in females; NTV and NTC: number of females dropped from the immunized and control groups of animals, respectively.


DO (%)=100 (1−PATV/PATC),

DO: reduction in egg masses; PATV and PATC: mean weight of egg masses of females fed on immunized and control group of animals, respectively.


DR (%)=100 (1−PMTV/PMTC),

DR: reduction in mean weight of adult females; PMTV and PMTC: mean weight of adult females dropped from the immunized and control groups of animals, respectively.


RF (%)=100 (1−RIV/RIC),

RF: reduction in adult fertility; RIV and RIC: mean RI of adult females dropped from the immunized and control groups of animals, respectively.


Reproductive Index (RI)=Egg masses/engorge tick weight.

E (%)=100 [1−(CRT×CRW×CRI)].

E%: Percentage efficacy of antigens. CRT = NTV/NTC, CRW = PMTV/PMTC, and CRI= RIV/RIC is the reduction in tick fertility.

### 2.8 Monitoring of immune responses

From all the animals, 2 ml of blood was collected aseptically during pre-immunization, immunization and post-immunization periods at different time intervals [pre-immunization, 17^th^, 27^th^, 40^th^ 49^th^, 62^nd^, 102^nd^ 119^th^ and 139^th^ day]. The serum was separated and stored at -20°C for further analysis as described earlier (39). Briefly, each microwell was coated with 1μg/ml concentration of each of rHaFER2 and rHaTPM, sera dilution 1:800 for IgG and 1:400 for IgG2, and the secondary antibodies was diluted to 1:10,000. Peroxidase mediated color development was measured at 492 nm in an ELISA reader (Tecan, Austria). The mean optical density (OD) was calculated for each time point by grouping control and immunized animals.

### 2.9 Statistical analysis

The one-way analysis of variance (ANOVA) with *post-hoc* Tukey HSD (Honestly Significant Difference) was used for analysis of differences at the gene transcript level, comparing the mean variation of entomological data of ticks fed on immunized group in comparison to control group of animals and for the mean antibody responses of calves in experimental and control groups at different time points. Significance at 5% level (*p*≤ 0.05) was used to define differences in different parameters.

## 3 Results

### 3.1 Double gene silencing effect on expression profile of the genes and its impact on feeding, survival and reproductive parameters of *H. anatolicum*

**Figure 1:**
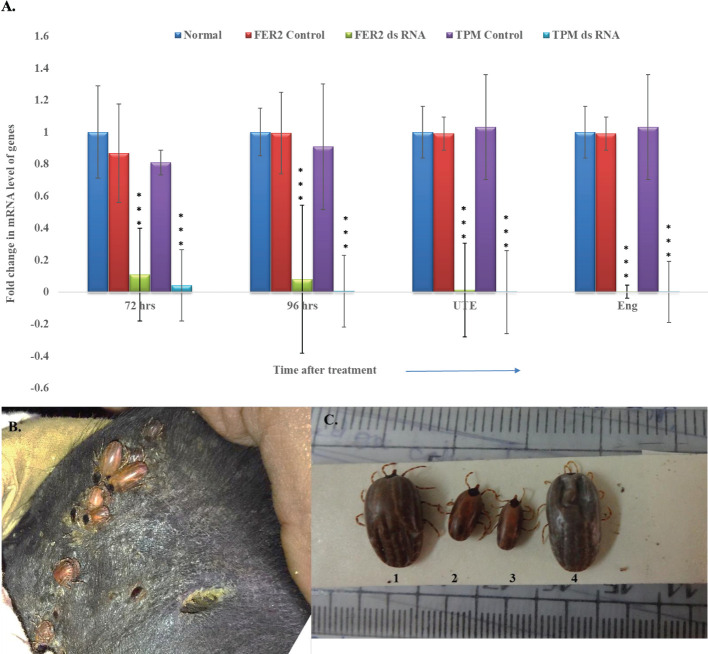
Post-double gene silencing effect on *Hyalomma anatolicum* IVRI-II strain. **(A)** Relative quantity of *FER2* and *TPM* genes transcript in dsRNA injected ticks vs normal and control (*Luc*-ds RNA) ticks [data were considered significantly different when *p*-value ≤0.05, within the treatment group & between treatment and control group (*p*<0.001), Error bars represent the variability in gene expression across multiple replicates.UTE- Unable to engorge, Eng-engorged tick. A notable decrease in gene expression was observed at both 72 and 96 hours post treatment, indicating a sustained and progressive effect of gene silencing. Luc-dsRNA as a control shows no significant effect on gene expression, confirming the specificity of the gene silencing effect in the FER2 and TPM dsRNA-treated groups] **(B)** Depicting *FER2&TPM* dsRNA injected *H. anatolicum* ticks attached on animal ear pinna. **(C)** Representative tick samples of *Luc*- dsRNA injected (No. 1 & 4), *FER2&TPM* dsRNA injected, ticks (No. 2 & 3) depicting change in tick size, shape and color of ticks.

A statistically significant (p < 0.001) reduction in expression of *FER2* and *TPM* genes in the dsRNA inoculated ticks at 72, 96 hr of feeding, in UTE and in the engorged ticks were noted in comparison to Luc control group and in normal ticks (**Figure 1A**). A consistent suppression of *FER2* and *TPM* genes was recorded throughout the feeding period.

Physically, silenced ticks were smaller in size, and discolored compared to the control group of ticks (**Figures 1B, C**). In silenced ticks, a remarkable 75.3 and 49.5% reduction in the number of engorged ticks and engorgement weight, respectively, were recorded. Interestingly, silenced ticks laid comparatively lesser egg masses than the control group of ticks and a 32.7% of the challenged ticks were UTE (**Table 1**).

**Table 1:** Double gene silencing effect on the biology of *H. anatolicum*.

ds RNA group	LUC gene control	double gene silencing (FER2+ TPM)
Total No. of ticks injected	95	75
Total No. of ticks released on animals	83	67
Percentage of ticks engorged	58.8 ± 9.0	23.4 ± 5.9***
Engorged weight (mg)	447.8 ± 12.05	225 ± 22.3***
Reduction in body weight (%)	–	49.5%
Egg masses (mg)	225.9 ± 13.2	10.4 ± 7.3***
Reduction in egg masses (%)	–	95.4%
Percentage of ticks unable to engorge (UTE)	2.8 ± 3.7	32.7 ± 6.5
Percentage of ticks rejected	32.9 ± 12.0	42.5 ± 3.2
Total reduction in ticks (%) (Rejection +UTE) ± SE	35.0 ± 8.6	75.3 ± 8.2***

***p<0.001 (in comparison to *Luc* control).

**Figure 2:**
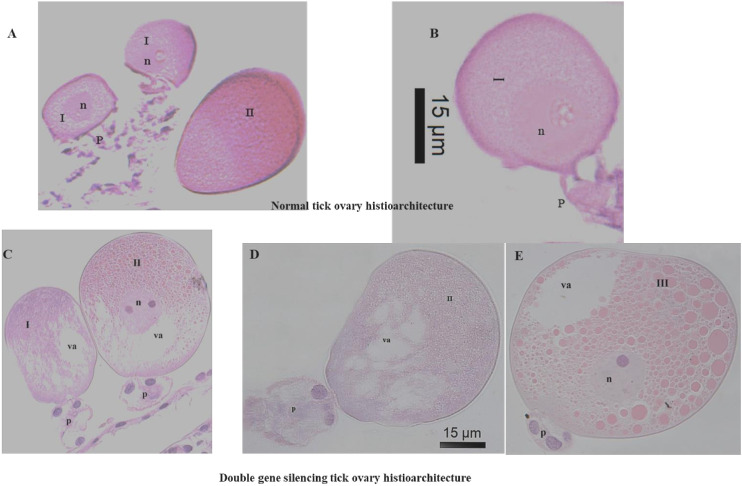
Comparative cellular changes in ovary (H&E stained) of control (*Luc* dsRNA) and double gene (*FER2*&*TPM*) silenced *Hyalomma anatolicum* ticks [Panels **(A, B)** are control, **(C–E)** are double gene silenced ovaries. I, oocyte type-I; II, oocyte type-II; III, oocyte type-III; va, vacuolation; n, nucleus; p, pedicle].

Sections of the ovary of silenced ticks revealed damaged plasma membrane, along with vacuolations at the periphery and at the oocyte pedicel junction of type I and II oocytes (**Figures 2C, D**) in comparison to the tissue sections of normal tick ovary (**Figures 2A, B**). In type III oocytes, the vacuolated areas were prominent near the germinal vesicles (**Figure 2E**). Most of the yolk granules in type III oocytes were negatively stained with hematoxylin and vacuolations were observed at the periphery (**Figure 2E**).

### 3.2 Protein expression, localization of native proteins

**Figure 3:**
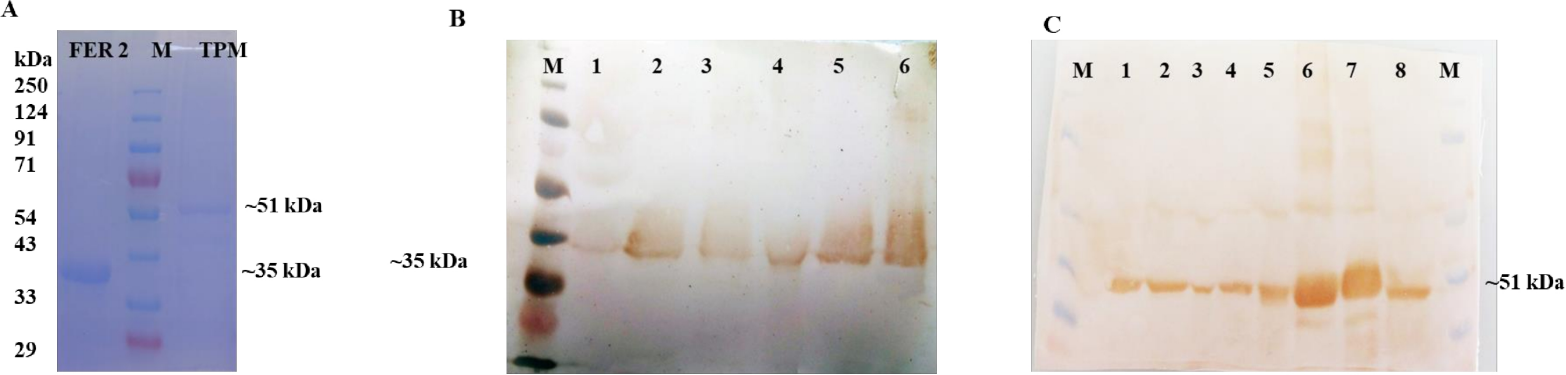
Expression and Western blot analysis of FER2 and TPM proteins. **(A)** SDS-PAGE profile of purified rHaFER2 and rHaTPM proteins of *Hyalomma anatolicum*; **(B)** Native FER2 in different developmental stages of *H. anatolicum* probed by rHaFER2 specific sera from rat. [M- Puregene marker, 1- egg, 2- Larvae, 3- Unfed nymph, 4- Fed nymph, 5- Unfed female, 6- Fed female]; **(C)** Native TPM in different developmental stages of *H. anatolicum* probed by rHaTPM specific sera from rat. [M- Puregene marker, 1- Larva, 2- Unfed nymph, 3- Fed nymph, 4- Unfed female, 5- Fed female, 6 & 7-rTPM, 8- Egg. and rHaTPM].

The predicted mature protein coding sequence of *FER2* and *TPM* were expressed in E.cloni^®^ chemically competent cells (Lucigen, USA) as ~35 kDa and 51 kDa fusion proteins, including 6x His tag. The expressed proteins were purified as rHaFER2 and rHaTPM (**Figure 3**) and used for raising anti-rHaFER2 and rHaTPM sera in rats. The native FER2 and TPM proteins were detected in eggs, larvae, unfed nymphs, engorged nymphs, unfed adults and in engorged ticks (**Figure 3**).

### 3.3 Immunization dose

The dose was determined based on number of fold rise in total IgG levels at maximum dilution (1: 12,800) of sera among the three groups of rats immunized with different doses of cocktail of proteins and was statistically analyzed. At the highest dilution (1: 12,800), a significant difference (p<0.001) was recorded in group II in comparison to group I and III animals. The antibody response in all the three immunized groups was significantly higher in comparison to the control group (**Table 2**). Accordingly, the immunization dose for *in vivo* trial was selected as 300 µg TD of each antigen.

**Table 2:** Comparison of mean IgG response in rats following inoculation with a cocktail of rHaFER2 and rHaTPM proteins in comparison to control group.

Weeks of serum collection	Group I (TD 150 µg each antigen) Mean OD ± SE	Group II (TD 300µg each antigen) Mean OD ± SE	Group III (TD 450 µg each antigen) Mean OD ± SE	Control Group Mean OD ± SE
Before 2^nd^ booster	0.103 ± 0.0013	0.217 ± 0.0015	0.165 ± 0.0024	0.16 **±** 0.016
1^st^ week of 2^nd^ booster	0.152 ± 0.0018	0.591 ± 0.0083	0.384 ± 0.0043	0.162 **±** 0.012
2^nd^ week of 2^nd^ booster	0.144 ± 0.0020	0.428 ± 0.0043***	0.381 ± 0.0066	0.106 ± 0.0002

*** Significant at *p*<0.0001 in comparison to Group I and Group III animals on 6^th^ week.

### 3.4 Immunization efficacy against challenge infestations

The experimental calves were active, feed and water intake were normal. The body temperatures were within the normal range, and no tick-borne infections were noticed in blood smear examinations throughout the experimental period of 139 days (data not shown).

Against *H. anatolicum*: Within 48 h of release, ticks started feeding on animals. Most of the engorged larvae dropped within 2 days of initiation of dropping from the control animals, while dropping of engorged larvae from the immunized animals lasted 4–6 days after initiation. Significant (*p <*0.01) reductions were noted in the dropping (43.16%) and molting (48.48%) percentages of larvae fed on the immunized group of animals in comparison to larvae fed on the control animals. The comparative post-challenge parameters, viz., the percentage reduction in the number of dropped larvae (DT %), the molting percentage (MO %), and the efficacy against larvae (E %) were determined as 43.16%, 48.46%, and 70.7%, respectively (**Table 3A**). No statistical differences were observed in the mean number of adult ticks dropped from the immunized and control groups of animals. However, a significant (*p <*0.001) reduction of 43.09% in the mean engorgement weight and egg masses (62.29%) laid by ticks fed on the immunized group of animals in comparison to ticks fed on the control group of animals were observed. The DT%, DR%, DO%, RF%, and E% were determined as 5.4%, 29.9%, 66.8%, 56.2%, and 76.2%, respectively (**Table 3B**).

Table 3Mean± SE efficacy of of calves immunized by the cocktails of rHaFER2 and rHaTPM proteins against challenge infestations of *H. anatolicum* IVRI-II larvae (A), adults (B) and *R. microplus* ticks (C).

**Table:** 

(A)						
Challenge Infestation	Group	No. of engorged larvae dropped	No. of larvae moulted to nymphs	DT%	MO%	E%
*H. anatolicum* larvae	Immunized	88.67 ± 4.2**	56± 3.60**	43.16	48.46	70.70
	Control	156 ± 16.3	108.7 ± 7.4			
	Reduction %	43.16	48.48

**Table:** 

(B)										
	Group	Tick dropped/animal	Tick wt. (mg)	Egg wt.(mg)	RI	DT%	DR %	DO %	RF%	E%
*H. anatolicum* adults	Immunized	5.75 ± 0.48	240.2 ± 11.1***	92.8 ± 11.08***	0.38 ± 0.05***	5.4	29.9	66.8	56.2	76.2
	Control	8.25 ± 0.63	422.1± 14.7	246.1 ± 10.02	0.58 ± 0.01					
	Reduction %	33.5	43.1	62.3	35.3

**Table:** 

(C)										
*R. microplus larvae*	Group	Tick Dropped/ Animal	Tick wt. (mg)	Egg wt.(mg)	RI	DT%	DR %	DO %	RF%	E%
	Immunized	51.67 ± 8.8	96.46 ± 4.0*	47.43 ± 3.4*	0.49 ± 0.02*	18.1	24.8	42.4	24.7	53.6
	Control	63.33 ± 2.3	131.1 ± 7.2	84.12 ± 3.33	0.65 ± 0.01					
	Reduction %	18.94	26.4	43.61	24.3					

Significant at ** *p <*0.01 *** *p <*0.001 in comparison to the control group of animals.

Against *R. microplus*: The challenged larvae started feeding within 48 hrs of release. After 18–19 days of feeding, engorged adults started to drop from all the animals. Significant reductions in the mean engorgement weight (*p <*0.005) and mean egg masses (*p <*0.05) of ticks collected from immunized group of animals in comparison to the control group of animals were noted. Consequently, due to the significant reduction in egg masses, the reproductive index was significantly (*p <*0.05) reduced by 43.61%. The DT%, DR%, DO%, RF%, and E% were determined as 18.1%, 24.8.0%, 42.4%, 24.7%, and 53.6%, respectively (**Table 3C**).

### 3.5 Antibody responses

**Figure 4:**
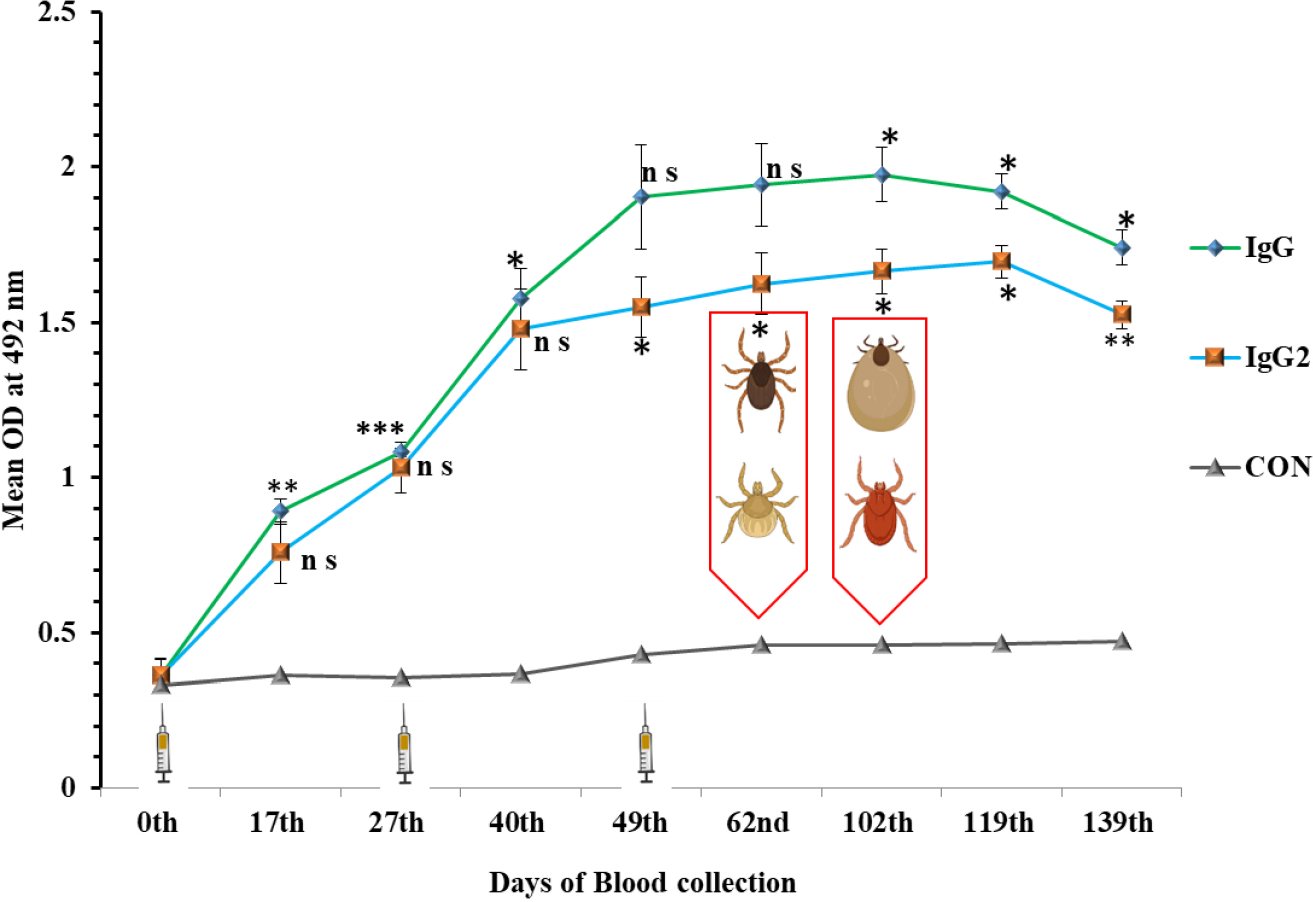
Immune response in cross- bred bovine calves after immunization with cocktail (rHaFER2 &rHaTPM) of proteins. Mean IgG and IgG2 response in calves immunized with cocktail of rHaFER-2 and rHaTPM proteins and control groups. (Syringe depicts time of immunization followed by two booster doses. Tick depicts the time of Ist and 2^nd^ challenge with *Hyalomma anatolicum* and *Rhipicephalus microplus* larvae after immunization. [ns, not significant; *p <0.05; **p <0.01; ***p <0.001 in comparison to the control group].

The immunization of animals stimulated the production of antibodies as early as 17^th^ day post primary immunization (DPI), and the significant response persisted until the end of the experiment (139^th^ DPI) (**Figure 4**). The mean optical density (OD) values of the sera collected on 17^th^ and 49^th^ DPI were 0.89 ± 0.04 and 1.90 ± 0.06, for IgG and 0.75 ± 0.02 and 1.57 ± 0.07 for IgG2, respectively, which was nearly 2.4-4.4 times higher than the control group of animals. After 120 DPI, a slight drop in IgG response was observed but the value was still 3.6 times higher than the pre-immunization value (**Figure 4**). Similarly, the IgG2 level rose from 0.36 ± 0.4 to 1.69± 0.12 in the immunized group of animals, which is almost 5-6 times higher in comparison to pre-immunization response and sustained till 139^th^ DPI (**Figure 4**). The IgG and IgG2 level titer remain at the base threshold level throughout the experimental period in control group of animals (**Figure 4**).

## 4 Discussion

Globally, TTBDs represent a growing burden with both re-emerging and newly emerging diseases posing threats to human and animal health (18). India, with the world’s largest population of 192.52 million cattle and 109.85 million buffaloes is facing significant challenges in maintaining the dairy industry in a sustainable manner due to the perennial tick infestation problems (47). Since more than four decades, the management of TTBDs has been focused on repeated applications of acaricides and consequently, several reports of multi-acaricide resistance *R. microplus* have been published globally (12, 48–55). Even though limited information is available on acaricide resistance in *H. anatolicum* (13, 56, 57), increasing level of resistance in *H. anatolicum* ticks collected from CCHF outbreak states of India viz., Gujarat and Rajasthan complicating the management of CCHFV infection in humans (54, 57).

Inducing host immunity through vaccines against ticks and tick-borne pathogens is a cost-effective and efficient strategy to reduce the use of chemical pesticides and the risk of establishment and spread of acaricide resistant ticks (12, 23, 35). Immunization strategy, a key component of integrated tick management, was proved effective by the commercialization of TickGARD™ and Gavac™ vaccines in the 1990s for controlling *R. microplus* (33, 58). Subsequently, new protective antigens were identified and characterized to manage multi-tick infestations and pathogen transmission (22, 23, 35). However, screening for highly effective antigens with cross-protective effects remains the main challenge in anti-tick vaccine research and development. Researchers confirmed that targets identified using *in-silico* analysis and RNAi technology are suitable for inclusion in multi-antigen vaccines (22, 59).

As an obligatory blood-sucking ectoparasite, ticks have evolved a set of special survival mechanisms, including long duration of blood sucking, preventing blood coagulation in the body, and storing blood meals in the midgut (35). The anti-tick vaccines are targeted to develop strong antibody responses to the targeted antigens and ingesting antibodies by the feeding tick will interact with the target proteins in the tick and disrupt feeding, molting, and reproduction (23, 35, 60). Previously, silencing of *FER2* and *TPM* genes in tick resulted in a reduction of tick infestation (rejection + unable to engorged tick) by 61.3% and 70.2%, respectively, in comparison to *LUC*-gene control group was recorded (39). In the present study, dual gene silencing resulted in a higher reduction of 75.3% of the injected ticks fed on immunized calves. Only 23% of the gene silenced ticks engorged, and the egg-laying capacity was severely impacted (**Table 1**). The data clearly shows a synergistic property of the targets, thus, its suitability in cocktail vaccine development. Several studies have demonstrated the synergistic effect of double gene silencing in different tick species. For example, de la Fuente et al. (61) demonstrated that silencing of Subolesin (*4D8*) and *Rs86* genes of *R. sanguineus* ticks, resulted in 97% reductions in engorgement, 87% in tick weight, 41% survival rate and 100% oviposition. Rahman et al. (62) conducted an RNAi trial using Subolesin and Cystatin genes in *Haemaphysalis longicornis* and a significant reduction in egg masses and egg conversion ratio were recorded. Further, in the present study, the gene silencing effect on the ovary of the injected ticks were studied to understand the possible reasons for cessions of egg laying/reduction in egg mass production in injected ticks. Ultra-thin sections of the ovary of dsRNA injected ticks revealed damage of plasmic membrane and vacuolations in the different stages of ovaries compared to the ovary of control ticks (**Figures 2C–E**), as reported in the ovary of the acaricide-treated ticks (44, 63). Since the ovaries of ticks were processed immediately after dropping from the animals, mostly 1^st^ and 2^nd^ stage oocytes were seen and the 3^rd^ stage oocytes were observed occasionally. The presence of native FER-2 and TPM proteins in all the developmental stages of *H. anatolicum* ticks was confirmed through western blot analysis using specific anti-FER-2 and anti-TPM sera raised in rats immunized with the respective recombinant proteins. The data was consistent with the observation of Galay et al. (64), who demonstrated the presence of FER-2 protein in whole ticks and in the midgut of *H. longicornis*. The present study also indicated that the recombinant proteins, despite being expressed in a prokaryotic expression system, underwent minimal post-translational modifications and confirmed that, the antigens are expressed in all the developmental stages of the *H. anatolicum*.

Globally, multiple tick research groups have followed different strategies with varying levels of efficacy against challenge infestations. These include i), up to 70% efficacy using cocktail of antigens (29–32); (ii) up to 90% efficacy by co-immunization with two or more protective antigens (31, 65, 66); and (iii) >60% efficacy using chimeric antigens as vaccines (67). Conversely, a single antigen immunization trial provided 64% protection against *R. microplus* challenge infestation in cattle following immunization with *R. microplus* Ferritin2 (RmFER2) (36). Previously, we reported 51.7% and 63.7% protection against challenged larvae while 51.2% and 66.4% protection against challenged adults of *H. anatolicum*, respectively, following immunization of cross-bred calves with *H. anatolicum* rHaFER2 and rHaTPM, respectively (39). It was hypothesized that the cocktail vaccine consisting of multiple tick antigens can have better cross-protection potential (28, 30). Subsequently, Schetters et al. (31) recorded 79% efficacy of Bm86 based vaccine against challenge infestation of *R. microplus* and *R. annulatus* whereas, in combination with Subolesin recorded an efficacy of 97%. Similarly, Almazan et al. (29) developed a cocktail formulation containing the *Ixodes scapularis* 4D8, 4F8, and 4E6 antigens and achieved an overall efficacy of 71%, with a 12-50% reduction in infestation and a 22-49% decrease in egg mass production. In an another study, Imamura et al. (68) reported a 61.4% reduction of engorgement of the challenged nymphs and an increase 28% of female and 43% male mortality using a cocktail of antigens containing *R. appendiculatus* serine protease inhibitors RAS-1 and RAS-2. Parizi et al. (30) formulated a cocktail of antigens comprising of Vitellin-degrading cysteine endopeptidase (VTDCE) and yolk pro-cathepsin (BYC) from *R. microplus* and Glutathione S- transferase of *H. longicornis* (GST-Hl). Immunization of animals resulted in a 61.6% reduction in overall tick population (30). Additionally, the cocktail vaccine composed of multiple parasite antigens demonstrated high protection levels against various parasites (68–71).

In the present study, immunized and control animals were challenged with larvae and adults of *H. anatolicum* and larvae of *R. microplus*. While larvae and adults of *H. anatolicum* immediately attached upon release to control animals, a notable delay in the attachment of challenged ticks was observed in the immunized animals, resulted in a mean efficacy of 70.7 and 76.2% against *H. anatolicum* larvae and adults, respectively. The delayed feeding could be attributed to the localization of TPM antibodies in the salivary glands and muscle fibers of various organs, and presence of ferritin2 antibodies in the gut and other tissues. The results showed a significant 43.09% reduction in the mean engorgement weight of adults followed by 62.29% reduction in mean egg masses laid by ticks fed on the immunized group of animals compared to the control group of animals (**Table 3**).

The efficacy of an immunogen depends not only on its type and quality of the antigens and adjuvant used but also on external factors, such as environmental conditions. Environmental factors like temperature, humidity, and tick population dynamics can significantly impact the vaccines performance. For instance, high temperatures may stress ticks, altering their feeding behavior and survival rates, while humidity affects tick activity and reproduction. Variations in tick population dynamics, including density and species composition, can also influence vaccine effectiveness. Fluctuations in local tick populations, driven by environmental changes or seasonal shifts, may alter exposure levels, thereby affecting the immune response in vaccinated cattle (17, 22, 33).

The cross-protection against *R. microplus* was comparatively less (53.6%) in comparison to protection observed against *H. anatolicum* but the protection data was comparable with the results obtained by different researchers using other antigens against heterologous challenge (19, 20, 72). The variations in cross-protective efficacy may be due to i) the differences in shared epitopes between *H. anatolicum* and *R. microplus* that can affect how the immune system recognizes and protects against different tick species, and ii) similar antigens may enhance protection through cross-reactive immune responses, while more distantly related species might elicit weaker immune reactions. To improve the relatively low cross-protection (53.6%) against *R. microplus*, we proposed some modifications in the experimental design to be taken up in the future research work. For example; i. Identification of better delivery system using engineered adjuvant capable to stimulate both humoral and cellular responses and exploration of alternative delivery routes, including intranasal and transdermal; ii. Identification and incorporation of additional or modified antigens that are conserved between different strains of *H. anatolicum* and *R. microplus* through epitope mapping; iii. Optimizing antigen combinations; iv. Implementing a prime-boost strategy to strengthen the immune response; and, v. Conducting longitudinal studies to evaluate the duration of protection.

Immunoglobulin G (IgG) is a key class of antibodies that includes four subclasses: IgG1, IgG2, IgG3, and IgG4. Each subclass has unique properties and functions. Carvalho et al. (73) reported an association between certain haplotypes of IgG2 with phenotypes of *R. microplus* infestations in cattle. Therefore, level of IgG2 may be indicated as development of tick resistance in host. In the present study, we analyzed both IgG and IgG2 to gain a thorough understanding of the immune response, as each provides important insights into different facets of antibody activity. Numerous immunization studies have established the role of the humoral antibody response in conferring immunity against ticks (30, 58, 65, 72). Accordingly, IgG and IgG2 antibody response was monitored by indirect ELISA. The mean OD values of sera collected after the second booster dose were 1.90 ± 0.06 and 1.54 ± 0.02 for IgG and IgG2, respectively, which were nearly >4.4 times higher than those in the control group of animals. Other researchers also reported a similar trend of high IgG antibody titers against Bm86 and Subolesin cocktail antigens in the immunization trial (31). De la Fuente et al. (33) reported an antibody titer cutoff of more than 1:640, indicating a damaging effect mediated by specific antibodies on ticks. The cocktail immunization successfully produced a higher antibody response against dual antigens (titer of 1: 6400) which impacted on the overall reduction of engorgement percentage, egg masses and reproductive index of ticks fed on immunized ticks. Besides, the damage observed in the ovary has limited the overall production of egg masses of ticks fed on immunized animals. The antibodies against TPM antigen are preferably bound to various muscle tissues in ticks, which impaired the feeding and egg laying capacity, possibly through complement-mediated lysis of the digestive cells of the challenged ticks. Although the current study was conducted with a minimum statistically significant number of cross-bred calves, the results will boost the confidence of researchers in developing a cocktail vaccine for the protection of animals against multi-tick infestations considering personalized vaccinology approaches and international collaborations (74).

In conclusion, the cocktail of HaFER2 and HaTPM antigens, combined with Montanide ISA 50V2 as an adjuvant, exhibited significant efficacy of 76.2% and 53.6% protection against *H. anatolicum* and *R. microplus* adult ticks. The protection was significantly correlated with the production of IgG response, highlighting the potential of FER2 and TPM antigens as promising candidates for developing a cocktail vaccine against these tick species. However, validation of the research output in field trials presents numerous challenges, such as environmental variability, animal health differences, and variability in tick-host interactions dynamics due to differences in frequency and intensity of tick bites in different management system. All of which can play role in vaccine efficacy. Additionally, logistical issues such as regulatory compliance and scalability needs to be addressed to achieve the optimum efficacy of immunization protocol in the filed situations in a sustainable manner.

## Data Availability

The raw data supporting the conclusions of this article will be made available by the authors, without undue reservation.

## References

[1] Jones KE Patel NG Levy MA Storeygard A Balk D Gittleman JL. Global trends in emerging infectious diseases. Nature. (2008) 451:990–3. doi: 10.1038/nature06536 PMC5960580 18288193

[2] de la Fuente J Estrada-Peña A Venzal JM Kocan KM Sonenshine DE. Overview: ticks as vectors of pathogens that cause disease in humans and animals. Front Biosci. (2008) 13:6938–46. doi: 10.2741/3200 18508706

[3] Ghosh S Azhahianambi PA Yadav MP. Upcoming and future strategies of tick control: a review. J Vector Borne Dis. (2007) 44:79. 17722860

[4] Kumar B Manjunathachar HV Ghosh S. A review on Hyalomma species infestations on human and animals and progress on management strategies. Heliyon. (2020) 1:6. doi: 10.1016/j.heliyon.2020.e05675 PMC7726666 33319114

[5] Spengler JR Bente DA. Crimean–Congo hemorrhagic fever in Spain — New arrival or silent resident? N Engl J Med. (2017) 377:106. doi: 10.1056/NEJMp1707436 PMC5922251 28700846

[6] Mourya DT Yadav PD Patil DY Sahay RR Rahi M. Experiences of Indian Council of Medical Research with tick-borne zoonotic infections: Kyasanur Forest disease & Crimean-Congo haemorrhagic fever in India with One Health focus. Indian J Med Res. (2021) 153:339–47. doi: 10.4103/ijmr.IJMR_532_21 PMC8204825 33906997

[7] Manjunathachar HV Raut CG Tiwari P Chouksey V Barde PV Yadav PD. Crimean-Congo hemorrhagic fever virus prevalence in livestock of Jabalpur, Madhya Pradesh, Central India and its implications for public health. Res Vet Sci. (2024) 171:105243. doi: 10.1016/j.rvsc.2024.105243 38564980

[8] de la Fuente J Ghosh S Lempereur L Garrison A Sprong H Lopez-Camacho C Maritz-Olivier C. Interventions for the control of Crimean- Congo hemorrhagic fever and tick vectors. .NPJ Vaccines. (2024) 9:181. doi: 10.1038/s41541-024-00970-5 39353956 PMC11445411

[9] Lew-Tabor AE Valle MR. A review of reverse vaccinology approaches for the development of vaccines against ticks and tick-borne diseases. Ticks tick-borne Dis. (2016) 7:573–85. doi: 10.1016/j.ttbdis.2015.12.012 26723274

[10] Manjunathachar HV Saravanan BC Kesavan M Karthik K Rathod P Gopi M. Economic importance of ticks and their effective control strategies. Asian Pac J Trop Med. (2014) 4:S770–9. doi: 10.1016/S2222-1808(14)60725-8

[11] Agwunobi DO Yu Z Liu J. A retrospective review on ixodid tick resistance against synthetic acaricides: implications and perspectives for future resistance prevention and mitigation. Pestic Biochem Physiol. (2021) 173:104776. doi: 10.1016/j.pestbp.2021.104776 33771255

[12] Evans A Madder M Fourie J Halos L Kumsa B Kimbita E. Acaricide resistance status of livestock ticks from East and West Africa and *in vivo* efficacy of acaricides to control them. Int J Parasitol Drugs Drug Resist. (2024) 25:100541. doi: 10.1016/j.ijpddr.2024.100541 38761529 PMC11133915

[13] Shyma KP Kumar S Sharma AK Ray DD Ghosh S. Acaricide resistance status in Indian isolates of *Hyalomma anatolicum* . Exp Appl Acarol. (2012) 58:471–81. doi: 10.1007/s10493-012-9592-3 22760859

[14] Kumar R Sharma AK Ghosh S. Menace of acaricide resistance in cattle tick, *Rhipicephalus microplus* in India: Status and possible mitigation strategies. Vet Parasitol. (2020) 278:108993. doi: 10.1016/j.vetpar.2019.108993 31954273

[15] Dzemo WD Thekisoe O Vudriko P. Development of acaricide resistance in tick populations of cattle: A systematic review and meta-analysis. Heliyon. (2022) 1:8. doi: 10.1016/j.heliyon.2022.e08718 PMC8760414 35059516

[16] Graf JF Gogolewski R Leach-Bing N Sabatini GA Molento MB Bordin EL. Tick control: an industry point of view. Parasitology. (2004) 129:S427–42. doi: 10.1017/S0031182004006079 15938522

[17] Ndawula C. From bench to field: A guide to formulating and evaluating anti-tick vaccines delving beyond efficacy to effectiveness. Vaccines. (2021) 9:1185. doi: 10.3390/vaccines9101185 34696291 PMC8539545

[18] de la Fuente J Mazuecos L Contreras M. Innovative approaches for the control of ticks and tick-borne diseases. Ticks tick-borne Dis. (2023) 14:102227. doi: 10.1016/j.ttbdis.2023.102227 37419001

[19] Perez-Perez D Bechara GH MaChado RZ Andrade GM Del Vecchio RE Pedroso MS. Efficacy of the Bm86 antigen against immature instars and adults of the dog tick *Rhipicephalus sanguineus* (Latreille, 1806) (Acari: Ixodidae). Vet Parasitol. (2010) 167:321–6. doi: 10.1016/j.vetpar.2009.09.034 19836894

[20] Kumar B Murugan K Ray DD Ghosh S. Efficacy of rBm86 against *Rhipicephalus (Boophilus) microplus* (IVRI-I line) and *Hyalomma anatolicum anatolicum* (IVRI-II line) infestations on bovine calves. Parasitol Res. (2012) 111:629–35. doi: 10.1007/s00436-012-2879-9 22422293

[21] Parthasarathi BC Kumar B Nagar G Manjunathachar HV de la Fuente J Ghosh S. Analysis of genetic diversity in Indian isolates of *Rhipicephalus microplus* based on *bm86* gene sequence. Vaccines. (2021) 9:194. doi: 10.1007/s00436-012-2879-9 33652549 PMC7996562

[22] Kabi F Dhikusooka M Matovu M Mugerwa S Kasaija P Emudong P. Monitoring the Subolesin vaccine field trial for safer control of cattle ticks amidst increasing acaricide resistance in Uganda. Vaccines. (2022) 10:1594. doi: 10.3390/vaccines10101594 36298461 PMC9609280

[23] Abbas MN Jmel MA Mekki I Dijkgraaf I Kotsyfakis M. Recent advances in tick antigen discovery and anti-tick vaccine developmen. Int J MolSci. (2023) 24:4969. doi: 10.3390/ijms24054969 PMC10003026 36902400

[24] Dowall SD Carroll MW Hewson R. Development of vaccines against Crimean-Congo haemorrhagic fever virus. Vaccine. (2017) 35:6015–23. doi: 10.1016/j.vaccine.2017.05.031 PMC5637709 28687403

[25] de la Fuente J Kocan KM. Strategies for development of vaccines for control of ixodid tick species. Parasite Immunol. (2006) 28:275–83. doi: 10.1111/j.1365-3024.2006.00828 16842264

[26] Willadsen P. Tick control: thoughts on a research agenda. Veterinary parasitology. (2006) 138:161–8. doi: 10.1016/j.vetpar.2006.01.050 16497440

[27] Ghosh S Azhahianambi P de la Fuente J. Control of ticks of ruminants, with special emphasis on livestock farming systems in India: present and future possibilities for integrated control-a review. Exp Appl Acarol. (2006) 40:49–66. doi: 10.1007/s10493-006-9022-5 17004030

[28] Willadsen P. Antigen cocktails: valid hypothesis or unsubstantiated hope? Trends Parasitol. (2008) 24:164–7. doi: 10.1016/j.pt.2008.01.005 18342573

[29] Almazán C Kocan KM Blouin EF de la Fuente J. Vaccination with recombinant tick antigens for the control of *Ixodes scapularis* adult infestations. Vaccine. (2005) 23:5294–8. doi: 10.1016/j.vaccine.2005.08.004 16153760

[30] Parizi LF Reck J Oldiges DP Guizzo MG Seixas A Logullo C. Multi-antigenic vaccine against the cattle tick *Rhipicephalus (Boophilus) microplus*: a field evaluation. Vaccine. (2012) 30:6912–7. doi: 10.1016/j.vaccine.2012.08.078 22981764

[31] Schetters TPM Jansen T. Vaccine against Rhipicephalus Ticks. International Application Number: PCT/EP2014/056248 (2014). International Publication Number: WO2014/154847Al (2015). Available at: https://patentscope.wipo.int/search/en/detail.jsf?docId=WO2014154847.

[32] Olds CL Mwaura S Odongo DO Scoles GA Bishop R Daubenberger C. Induction of humoral immune response to multiple recombinant *Rhipicephalus appendiculatus* antigens and their effect on tick feeding success and pathogen transmission. Parasite Vectors. (2016) 9:1–1. doi: 10.1186/s13071-016-1774-0 PMC5010713 27589998

[33] de la Fuente J Rodríguez M Redondo M Montero C García-García JC Méndez. Field studies and cost-effectiveness analysis of vaccination with Gavac against the cattle tick *Boophilus microplus* . Vaccine. (1998) . 16:366–73. doi: 10.1016/s0264-410x(97)00208-9 9607057

[34] Vaughan JA Sonenshine DE Azad AF. Kinetics of ingested host immunoglobulin G in hemolymph and whole-body homogenates during nymphal development of *Dermacentor variabilis* and *Ixodes scapularis* ticks (Acari: Ixodidae). Exp Appl Acarol. (2002) 27:329–40. doi: 10.1023/A:1023347930746 12797408

[35] Chen L Chen H Yue C Ma R Fan X Liu S. Evaluation of the immunoprotective effect of prokaryotic expression of *Haemaphysalis fulvae* tick salivary gland protein and ferritin 1. J Anim Husb Vet Med. (2024) 55:688–97.

[36] Hajdusek O Almazán C Loosova G Villar M Canales M Grubhoffer L. Characterization of ferritin 2 for the control of tick infestations. Vaccine. (2010) 28:2993–8. doi: 10.1016/j.vaccine.2010.02.008 20171306

[37] Galay RL Aung KM Umemiya-Shirafuji R Maeda H Matsuo T Kawaguchi H. Multiple ferritins are vital to successful blood feeding and reproduction of the hard tick. Haemaphysalis longicornis. J Exp Biol. (2013) 216:1905–15. doi: 10.1242/jeb.081240 23393286

[38] Ranjbar MM Gupta SK Ghorban K Nabian S Sazmand A Taheri M. Designing and modeling of complex DNA vaccine based on tropomyosin protein of *Boophilus* genus tick. Appl Biochem Biotechnol. (2015) 175:323–39. doi: 10.1007/s12010-014-1245-z 25269597

[39] Manjunathachar HV Kumar B Saravanan BC Choudhary S Mohanty AK Nagar G. Identification and characterization of vaccine candidates against *Hyalomma anatolicum*—Vector of CrimeanCongo haemorrhagic fever virus. Transbound Emerg Dis. (2019) 66:422–34. doi: 10.1111/tbed.13038 30300470

[40] Tian M Tian Z Luo J Xie J Yin H Zeng Q. Identification of the tropomyosin (HL-Tm) in *Haemaphysalis longicornis* . Vet Parasitol. (2015) 207:318–23. doi: 10.1016/j.vetpar.2014.10.007 25535026

[41] Ghosh S Azhahianambi P. Laboratory rearing of *Theileria annulata*-free *Hyalomma anatolicum anatolicum* ticks. Exp Appl Acarol. (2007) 43:137–46. doi: 10.1007/s10493-007-9100-3 17851768

[42] Nijhof AM Taoufik A de la Fuente J Kocan KM De Vries E Jongejan F. Gene silencing of the tick protective antigens, Bm86, Bm91 and subolesin, in the one-host tick *Boophilus microplus* by RNA interference. Int J Parasitol. (2007) 37:653–62. doi: 10.1016/j.ijpara.2006.11.005 PMC1885961 17196597

[43] Kumar B Manjunathachar HV Nagar G Ravikumar G de la Fuente J Saravanan BC. Functional characterization of candidate antigens of *Hyalomma anatolicum* and evaluation of its cross-protective efficacy against *Rhipicephalus microplus* . Vaccine. (2017) 35:5682–92. doi: 10.1016/j.vaccine.2017.08.049 28911904

[44] Ajithkumar KG Fular A Chigure G Sharma AK Nagar G Souza FF. Comparative impact of coumaphos, amitraz and plant extract of *Ageratum conyzoides* on the oogenesis of *Rhipicephalus microplus* . Ticks Tick Borne Dis. (2019) 10:1085–95. doi: 10.1016/j.ttbdis.2019.06.003 31186201

[45] Manjunathachar HV Saravanan BC Kumar B Ghosh S. Expression and determination of immunization dose of recombinant tropomyosin protein of *Hyalomma anatolicum* for the development of anti-tick vaccine. Indian J Anim Res. (2017) 87:275–9. doi: 10.56093/ijans.v87i3.68841

[46] Ghosh S Khan MH. Isolation of immunoreactive proteins from the larval extracts of *Boophilus microplus* and *Hyalomma anatolicum anatolicum* . Indian J Anim Sci. (2000), 70.

[47] 20th Livestock Census-2019, All India Report. (2020). Department of Animal Husbandry and Dairying, ministry of fisheries animal husbandry and dairying. New Delhi: government of India (2019). p. 119. Available at: https://dahd.nic.in/ahs-division/20th-livestock-census-2019-all-India-report.

[48] Miller RJ Davey RB George JE. Modification of the food and agriculture organization larval packet test to measure amitraz-susceptibility against Ixodidae. J Med Entomol. (2002) 39:645–51. doi: 10.1603/0022-2585-39.4.645 12144297

[49] Li AY Davey RB George JE. Carbaryl resistance in Mexican strains of the southern cattle tick (Acari: Ixodidae). J Econ Entomol. (2005) 98:552–6. doi: 10.1093/jee/98.2.552 15889748

[50] Castro-Janer E Martins JR Mendes MC Namindome A Klafke GM Schumaker TT. Diagnoses of fipronil resistance in Brazilian cattle ticks (*Rhipicephalus (Boophilus) microplus*) using *in vitro* larval bioassays. Vet Parasitol. (2010) 173:300–6. doi: 10.1016/j.vetpar.2010.06.036 20688434

[51] Kumar S Paul S Sharma AK Kumar R Tewari SS Chaudhuri P. Diazinon resistant status in *Rhipicephalus (Boophilus) microplus* collected from different agro-climatic regions of India. Vet Parasitol. (2011) 181:274–81. doi: 10.1016/j.vetpar.2011.04.030 21600695

[52] Reck J Klafke GM Webster A Dall’Agnol B Scheffer R Souza UA. First report of fluazuron resistance in *Rhipicephalus microplus*: a field tick population resistant to six classes of acaricides. Vet Parasitol. (2014) 201:128–36. doi: 10.1016/j.vetpar.2014.01.012 24560364

[53] Ghosh S Kumar R Nagar G Kumar S Sharma AK Srivastava A. Survey of acaricides resistance status of *Rhipiciphalus (Boophilus) microplus* collected from selected places of Bihar, an eastern state of India. Ticks Tick Borne Dis. (2015) 6:668–75. doi: 10.1016/j.ttbdis.2015.05.013 26117183

[54] Gaur RS Sangwan AK Sangwan N Kumar S. Acaricide resistance in *Rhipicephalus (Boophilus) microplus* and *Hyalomma anatolicum* collected from Haryana and Rajasthan states of India. Ex. Appl Acarol. (2016) 69:487–500. doi: 10.1007/s10493-016-0046-1 27100113

[55] Klafke G Webster A Agnol BD Pradel E Silva J de la Canal LH. Multiple resistance to acaricides in field populations of *Rhipicephalus microplus* from Rio Grande do Sul state, Southern Brazil. Ticks Tick Borne Dis. (2017) 8:73–80. doi: 10.1016/j.ttbdis.2016.09.019 27717758

[56] Gupta S Sangwan N Sangwan AK Gupta S Kumar A Maan S. Acaricide resistance status of deltamethrin and coumaphos in *Hyalomma anatolicum* ticks collected from different districts of Haryana. Exp Appl Acarol. (2024) 92:809–33. doi: 10.1007/s10493-023-00894-0 38448756

[57] Singh NK Gelot IS Jyoti Bhat SA Singh H Singh V. Detection of acaricidal resistance in *Hyalomma anatolicum anatolicum* from Banaskantha district, Gujarat. J Parasit Dis. (2015) 39:563–6. doi: 10.1007/s12639-013-0397-z PMC4554566 26345072

[58] Kemp DH Pearson RD Gough JM Willadsen P. Vaccination against *Boophilus microplus*: Localization of antigens on tick gut cells and their interaction with the host immune system. Exp Appl Acarol. (1989) . 7:43–58. doi: 10.1007/BF01200452 2667918

[59] de la Fuente J Kocan KM Almazán C Blouin EF. RNA interference for the study and genetic manipulation of ticks. Trends Parasitol. (2007) 23:427–33. doi: 10.1016/j.pt.2007.07.002 17656154

[60] de la Fuente J Moreno-Cid JA Canales M Villar M de la Lastra JM Kocan KM. Targeting arthropod subolesin/akirin for the development of a universal vaccine for control of vector infestations and pathogen transmission. Vet Parasitol. (2011) 181:17–22. doi: 10.1016/j.vetpar.2011.04.018 21561715

[61] de la Fuente J Almazán C Naranjo V Blouin EF Kocan KM. Synergistic effect of silencing the expression of tick protective antigens 4D8 and Rs86 in *Rhipicephalus sanguineus* by RNA interference. Parasitol Res. (2006) 99:108–13. doi: 10.1007/s00436-006-0132-0 16518610

[62] Rahman MK Saiful Islam M You M. Impact of subolesin and cystatin knockdown by RNA interference in adult female *Haemaphysalis longicornis* (Acari: Ixodidae) on blood engorgement and reproduction. Insects. (2018) 9:39. doi: 10.3390/insects9020039 29614797 PMC6023342

[63] Roma GC Furquim KC Bechara GH Camargo Mathias MI. Cytotoxic effects of permethrin in oocytes of *Rhipicephalus sanguineus* (Acari: Ixodidae) fully engorged females: I. Direct or indirect action of the acaricide in germ cells? Exp Appl Acarol. (2011) 53:287–99. doi: 10.1007/s10493-010-9401-9 20960224

[64] Galay RL Umemiya-Shirafuji R Bacolod ET Maeda H Kusakisako K Koyama J. Two kinds of ferritin protect ixodid ticks from iron overload and consequent oxidative stress. PloS One. (2014) 9:e90661. doi: 10.1371/journal.pone.0090661 24594832 PMC3940913

[65] McKenna RV Riding GA Jarmey JM Pearson RD Willadsen P. Vaccination of cattle against the *Boophilus microplus* using a mucin-like membrane glycoprotein. Parasite Immunol. (1998) 20:325–36. doi: 10.1046/j.1365-3024.1998.00149.x 9717194

[66] Parthasarathi BC Kumar B Bhure SK Sharma AK Manisha Nagar G. Co-immunization efficacy of recombinant antigens against *Rhipicephalus microplus* and *Hyalomma anatolicum* tick infestations. Pathogens. (2023) 12:433. doi: 10.3390/pathogens12030433 36986356 PMC10058648

[67] Almazán C Moreno-Cantú O Moreno-Cid JA Galindo RC Canales M Villar M. Control of tick infestations in cattle vaccinated with bacterial membranes containing surface-exposed tick protective antigens. Vaccine. (2012) 30:265–72. doi: 10.1016/j.vaccine.2011.10.102 22085549

[68] Imamura S Namangala B Tajima T Tembo ME Yasuda J Ohashi K. Two serine protease inhibitors (serpins) that induce a bovine protective immune response against *Rhipicephalus appendiculatus* ticks. Vaccine. (2006) 24:2230–7. doi: 10.1016/j.vaccine.2005.10.055 16314008

[69] Wiśniewski M Łapiński M Daniłowicz-Luebert E Jaros S Długosz E Wędrychowicz H. Vaccination with a cocktail of *Ancylostoma ceylanicum* recombinant antigens leads to worm burden reduction in hamsters. Acta Parasitol. (2016) 61:556–61. doi: 10.1515/ap-2016-0074 27447220

[70] Picchio MS Sánchez VR Arcon N Soto AS Perrone Sibilia M Aldirico MLA. Vaccine potential of antigen cocktails composed of recombinant *Toxoplasma gondii* TgPI-1, ROP2 and GRA4 proteins against chronic toxoplasmosis in C3H mice. Exp Parasitol. (2018) 185:62–70. doi: 10.1016/j.exppara.2018.01.006 29309783

[71] Nisbet AJ McNeilly TN Price DRG Oliver EM Bartley Y Mitchell M. The rational simplification of a recombinant cocktail vaccine to control the parasitic nematode Teladorsagia circumcincta. Int J Parasitol. (2019) 49:257–65. doi: 10.1016/j.ijpara.2018.10.006 PMC6456928 30690091

[72] Rodríguez-Valle M Taoufik A Valdés M Montero C Hassan I Hassan SM. Efficacy of *Rhipicephalus (Boophilus) microplus* Bm86 against *Hyalomma dromedarii* and *Amblyomma cajennense* tick infestations in camels and cattle. Vaccine. (2012) 30:3453–8. doi: 10.1016/j.vaccine.2012.03.020 22446633

[73] Carvalho WA Ianella P Arnoldi FG Caetano AR Maruyama SR Ferreira BR. Haplotypes of the bovine IgG2 heavy gamma chain in tick-resistant and tick-susceptible breeds of cattle. Immunogenetics. (2011) 63:319–24. doi: 10.1007/s00251-011-0515-y PMC3068256 21301827

[74] de la Fuente J Gortázar C Contreras M Kabi F Kasaija P Mugerwa S. Increasing access to biotech products for animal agriculture in Sub-Saharan Africa through partnerships. Nat Biotechnol. (2024) 42:1013–4. doi: 10.1038/s41587-024-02300-5 38918615

